# Understanding the demographic and socioeconomic determinants of morbidity in Eastern Uganda: a retrospective analysis of the Iganga-Mayuge health and demographic surveillance data

**DOI:** 10.1136/bmjph-2024-000898

**Published:** 2024-11-28

**Authors:** Steve Bicko Cygu, Betty Nabukeera, Lindsey English, Shakira Babirye, Collins Gyezaho, Maureen Ng'etich, Michael Ochola, David Amadi, Henry Owoko Odero, Grace Banturaki, Damazo Twebaze Kadengye, Agnes Kiragga, Dan Kajungu

**Affiliations:** 1 Data Science Program, African Population and Health Research Center, Nairobi, Kenya; 2 Centre for Health and Population Research (MUCHAP), Iganga, Makerere University, Kampala, Uganda; 3 Department of Nutritional Sciences, University of Michigan School of Public Health, Ann Arbor, Michigan, USA; 4 Statistics, Infectious Diseases Research Collaboration, Kampala, Uganda; 5 Informatics and Data Science, The University of Manchester Faculty of Medical and Human Sciences, Manchester, UK; 6 Infectious Diseases Institute, Makerere University College of Health Sciences, Kampala, Uganda; 7 Department of Economics and Statistics, Kabale University, Kabale, Uganda; 8 Centre for Health and Population Research (MUCHAP), Makerere University, Kampala, Uganda; 9 Department of Global Health, Stellenbosch University Faculty of Medicine and Health Sciences, Cape Town, South Africa

**Keywords:** Public Health, Epidemiologic Factors, statistics and numerical data

## Abstract

**Introduction:**

Understanding the determinants of disease burden is imperative in enhancing population health outcomes. This study uses data from the Iganga-Mayuge Health and Demographic Surveillance Site, to understand demographic and socioeconomic factors influencing morbidity.

**Methods:**

We analysed secondary data from 2018 to 2023. We employed graphs and tables to present morbidity patterns across different sociodemographic factors and applied mixed-effects multinomial multivariate logistic regression model to understand the correlates of morbidity.

**Results:**

The findings reveal a predominant prevalence of malaria, lower respiratory tract infections, coryza, gastric acid-related and urinary tract infections, collectively constituting 83% of diagnosed diseases. Noteworthy demographic variations, particularly gender and age, significantly impact disease distribution, revealing higher diagnosis rates among females. Additionally, socioeconomic factors, including education and wealth status, contribute to discernible differences in disease burden.

**Conclusion:**

This research provides crucial insights into the implications of demographic and socioeconomic factors on disease burden in Uganda. The results contribute to evidence-based policy-making, highlighting the necessity for targeted interventions addressing specific health challenges encountered by diverse populations. The study advocates for continuous assessment of the epidemiological landscape to inform more tailored and effective health strategies, ultimately enhancing resilience in disease control efforts.

**WHAT IS ALREADY KNOWN ON THIS TOPIC**

- Health-related quality of life indicators vary significantly across different demographic and socioeconomic groups, particularly in low-resource settings such as Iganga-Mayuge. Socioeconomic determinants such as household income, education level and access to healthcare services are crucial factors influencing morbidity and health outcomes. Studies have shown that lower household incomes and limited access to education and healthcare are associated with higher rates of diseases such as malaria, HIV/AIDS and respiratory infections, contributing to disparities in health outcomes.

**WHAT THIS STUDY ADDS**

- This study provides a comprehensive analysis of the disease burden in periurban and rural Uganda, revealing the impact of demographic and socioeconomic differences on morbidity. It highlights the protective effect of higher education on health and the substantial gender differences in healthcare-seeking behaviours. The findings offer evidence-based insights for targeted interventions and policy strategies to address health inequities in these communities.

**HOW THIS STUDY MIGHT AFFECT RESEARCH, PRACTICE OR POLICY**

- For research, the study identifies critical areas such as gender disparities in healthcare-seeking behaviours and the protective effect of higher education on health outcomes. This will guide future studies. In practice, the findings emphasise the necessity of targeted health interventions and educational programmes tailored to specific population needs, particularly for women, children and individuals with lower educational attainment. From a policy perspective, the study advocates for increased investments in healthcare infrastructure in non-urban areas and initiatives to improve educational access, with an aim to reduce health disparities and promote equitable health outcomes.

## Introduction

 Improved population health is an essential part of a country’s well-being, economic development^1^ and citizen quality of life.^2^ Countries, therefore, have put in place incentives to improve population health through changes in healthcare access, targeted approaches to reduce disparities and investments in preventative health. Improvements in population health can reduce mortality^3 4^ and reduce the burden of disease.^5^

The 2021 Global Burden of Diseases, injuries and risk factors report highlighted differences in quality of health-related quality of life (HRQoL) across age, gender, location and socioeconomic groups.^6^ In low-resource settings such as Iganga-Mayuge, HRQoL indicators, which capture the broader impact of diseases on daily life, are particularly important as they reflect the real-life impact of health conditions amidst socioeconomic challenges.^7 8^ For instance, the burden of diseases such as malaria, HIV/AIDS and non-communicable diseases can be profound, affecting individuals’ ability to work, care for their families and participate in community life.

Determinants of morbidity include both demographics such as sex and age and socioeconomic indicators such as household income and access to health facilities.^9^ Across these determinants, there are known differences in disease distribution.^10^ For instance, studies have shown that those with lower household incomes experience greater morbidity, including malaria,^11^ HIV^12^ and respiratory infections.^13 14^ Research has also shown that risks for respiratory infections are higher in urban residents,^15^ while those in rural areas suffer a greater burden from malaria.^11^ Similar variability in disease burden was observed in a sample within sub-Saharan Africa (SSA), which also found worse health outcomes for those with lower maternal education and unsafe housing.^16^ Without consideration for these differences, country-wide changes to health systems will not improve health outcomes and will continue to widen the gap in health equity.^17^

Discrimination in healthcare settings contributes to the lack of family planning services being used by women^18^,^20^ and exacerbates the high morbidity and mortality related to female reproductive health.^21 22^ Education access is also a policy area that, with improvement, can improve health outcomes.^23^ While primary education in Uganda is free,^20^ costs related to transportation, food and boarding make access still difficult for many low-income families. Secondary education is not free, and familial support is needed for students to move through higher education.^24^ Globally, those with higher levels of education have improved health outcomes.^25 26^ The inequitable access to truly free, quality education in Uganda contributes to both income and health inequality.^27^

An in-depth understanding of the implications of demographic and socioeconomic differences on disease burden in Uganda is justified for many reasons. First, it will help address health inequities by identifying and understanding the specific health challenges faced by different populations. This knowledge can inform the development of targeted interventions and the allocation of resources to meet the unique needs of various age and gender groups. Second, the study findings can serve as a foundation for evidence-based policy-making, ensuring that policies are informed by robust data and tailored to address the identified disparities. Additionally, the study will contribute to the strengthening of health systems by improving data-driven decision-making processes and enhancing overall population health outcomes. Moreover, conducting such a study aligns with global health agendas and demonstrates Uganda’s commitment to achieving equitable healthcare. Thus, the aims of this study are to (1) analyse the differences in disease burden within those seeking healthcare in periurban and rural Uganda, (2) examine current policy influences on observed differences and (3) offer intervention and policy strategies to address disease disparities.

## Methods

### Study setting

The study was conducted within the population cohort of Iganga-Mayuge Health and Demographic Surveillance Sites (IMHDSS) located in eastern Uganda. The IMHDSS cohort covers a catchment area of 155 km^2^ with a population of 95 000 individuals and is served by 23 health facilities. Within the demographic surveillance area, which includes 2 general hospitals, 15 level II health centres (HC II) (11 public, 3 private not-for-profit (PNFP) and 1 private-for-profit), 6-level III HCs (5 public and 1 PNFP), 1 level IV HC (public) and over 20 private clinics. Censuses are conducted within the demographic surveillance area, either biannually or sometimes annually, to update information on various demographic events such as births, deaths, socioeconomic status, education, migration and more for all residents. The cohort characteristics have been profiled elsewhere.^28^

### Morbidity surveillance

In 2018, the Makerere University Centre for Health and Population Research, which runs the IMHDSS population cohort, established a morbidity surveillance system. This system facilitates the linkage of electronic health records (EHRs) at one community-based health centre (Busowobi HC III) with the households’ and residents’ characteristics. The EHR at Busowobi Health Centre III (HC III) captures diseases and their manifestation, uptake of vaccination and family planning services, antenatal and postnatal care information, all laboratory tests performed, drugs prescribed and dispensed. Individual and household information with individual unique identifier information for all members from the IMHDSS catchment area of Busowobi HC III was embedded into the EHR. Therefore, every member of the IMHDSS that visits the health facility is linked using that unique identifier, which facilitates the linkage of the two databases. A subset of the IMHDSS database with a total of 31 158 residents of 21 villages (the health facility catchment area) was preloaded and embedded on the EHR at Busowobi HC III. The linkage system allowed the team to correlate social demographic information collected at the household and community levels to the clinical data generated at the health facility.

### Data collection

Each patient in the surveillance area seeking care at Busowobi HC III was registered in the EHR system at the outpatient department, assigned a patient ID and their vitals captured. The EHR system records data in different tables: outpatient department, signs and symptoms, clinical diagnosis, laboratory test results, medicines prescribed and dispensed, family planning services, antenatal care clinic, maternity and immunisation tables. Consent to use this information beyond patient care was sought from all participants prior to capturing any information. Data collection started in August 2018 and is ongoing. For this study, we used data from August 2018 to June 2023.

### Data analysis

Morbidity prevalence was used to measure the burden of the top five leading diseases—calculated as the number of confirmed diagnoses for a particular disease divided by the total confirmed diagnoses for all the diseases. For example, if 50 individuals were diagnosed with Malaria out of 1000 individuals who were diagnosed with at least one condition, then the morbidity prevalence is 50/1000=5%. To account for reported variations in disease burden across demographics and socioeconomic variables, the proportional burden of diseases was delineated separately for each demographic and socioeconomic variable. This approach was adopted due to existing studies that have highlighted distinctions in disease impact based on demographic and socioeconomic factors.^1 2^ The age was categorised into four groups: 0–14 years, 15–24 years, 25–64 years and 65 and over; household size (defined as the number of members in a household) was categorised into three groups: less than 5 members, 5–9 members, 10 members and above; total number of visits (defined as the number of times the patients visited the hospital) was categorised into 3 groups: 1 visit, 2 visits, 3 visits and above. Other covariates used in the analysis include the following: gender (male, female), education level (none=no education, primary=primary education, secondary and above=past primary), place of residence (periurban, rural), wealth status (poor, rich—generated based on the household ownership of selected set of assets, such as televisions, bicycles, cars, dwelling characteristics, water, sanitation, and hygiene facilities,^29^ and year of diagnosis (2018–2023). We opted for categorisation of continuous variables such as age, household size and the number of visits to enhance both descriptive and analytical understanding. In particular, age categorisation allows for the exploration of age-group-specific patterns, for example, prevalence of morbidity in children; household size categorisation captures the potential differences disease associated exposure, for example, large households may face unique challenges in managing infectious diseases; and the number of visits categorisation can highlight the differences in health-seeking behaviours. Over 70 different diseases were diagnosed between 2018 and 2023. The top five leading diseases, which accounted for over 81% of the diagnosed diseases, were presented to show the most important sources of morbidity burden.

To understand the population and social determinants of morbidity, we fitted a mixed-effects multivariate multinomial logistic model (see online supplemental material 1). We applied a Bayesian approach to estimate the model parameters. All analyses were performed by using R software. The R package brms^30^ was used to fit the model.

## Results

### Descriptive findings

Between 2018 and 2023, a total of 10 166 visits were made to the health facility by individuals from the Iganga-Mayuge HDSS catchment area. These visits were made by 3765 unique individuals, the majority of whom were female (66%, n=2475), compared with males (34%, n=1290). The gender distribution was statistically significant (p<0.001). The largest age group among the patients was children and adolescents (0–14 years), accounting for 41% of the total population. Young adults (15–24 years) accounted for 22%, adults (25–64 years) accounted for 33% and the elderly (65+ years) accounted for 4.1%. The age distribution showed significant gender disparities (p<0.001). Nearly one-third of the patients (31%) had no formal education, with a higher percentage of males (33%) compared with females (30%) in the same category. Patients with primary education made up 45%, and those with at least secondary education accounted for 24%. The differences in educational levels across the genders were statistically significant (p=0.030). The majority of the patients (68%) lived in rural areas, with almost similar distributions among females (68%) and males (67%). Patients from periurban areas made up 32% of the total, with no clear gender differences across the residential areas (p=0.800). The wealth status of the patients showed that 71% were classified as poor, with almost equal distribution between females and males. Rich patients made up 29% of the total. The wealth status did not show clear differences between genders (p=0.600). Households with less than 5 members made up 11% of the patient population, while those with 5–9 members made up 50% and larger households (10+ members) accounted for 39%. The distribution of household sizes did not show clear differences between the genders (p=0.600). The majority of patients (47%) visited the healthcare facility only once, with a higher percentage of males (52%) making a single visit compared with females (45%). Patients making two visits accounted for 24%, and those with three or more visits accounted for 29%. The number of visits showed a significant gender difference (p<0.001). The distribution of patients by year of diagnosis varied, with 4.5% diagnosed in 2018, 10.0% in 2019, 27% in 2020, 29% in 2021, 29% in 2022, and 12% in 2023. There was no clear difference in the year of diagnosis between genders (p=0.200). Table 1 provides a summary of the study population.

**Table 1:** Baseline characteristics of patients seeking healthcare at Basowobi HC III

Characteristics	Total	Female	Male	P value*
	N (%)	n (%)	n (%)	
Patients	3765 (100%)	2475 (66%)	1290 (34%)	*<*0.001
Age groups (years)				*<*0.001
0–14	1544 (41%)	903 (36%)	641 (50%)	
15–24	826 (22%)	570 (23%)	256 (20%)	
25–64	1242 (33%)	903 (36%)	339 (26%)	
65+	153 (4.1%)	99 (4.0%)	54 (4.2%)	
Education level				0.030
None	1171 (31%)	739 (30%)	432 (33%)	
Primary	1697 (45%)	1151 (47%)	546 (42%)	
Secondary and above	897 (24%)	585 (24%)	312 (24%)	
Residence				0.800
Rural	2544 (68%)	1675 (68%)	869 (67%)	
Peri urban	1221 (32%)	800 (32%)	421 (33%)	
Wealth status				0.600
Poor	2691 (71%)	1763 (71%)	928 (72%)	
Rich	1074 (29%)	712 (29%)	362 (28%)	
Household size				0.600
*<*5	432 (11%)	281 (11%)	151 (12%)	
5–9	1865 (50%)	1216 (49%)	649 (50%)	
10+	1468 (39%)	978 (40%)	490 (38%)	
Number of visits				*<*0.001
1	1769 (47%)	1104 (45%)	665 (52%)	
2	911 (24%)	586 (24%)	325 (25%)	
3+	1085 (29%)	785 (32%)	300 (23%)	
Year of diagnosis				0.200
2018	170 (4.5%)	117 (4.7%)	53 (4.1%)	
2019	376 (10.0%)	245 (9.9%)	131 (10%)	
2020	1015 (27%)	636 (26%)	379 (29%)	
2021	659 (18%)	436 (18%)	223 (17%)	
2022	1089 (29%)	736 (30%)	353 (27%)	
2023	456 (12%)	305 (12%)	151 (12%)	

* Pearson’s Chi-squaredχ2 test.

HChealth centre

### Disease burden

**Figure 1:**
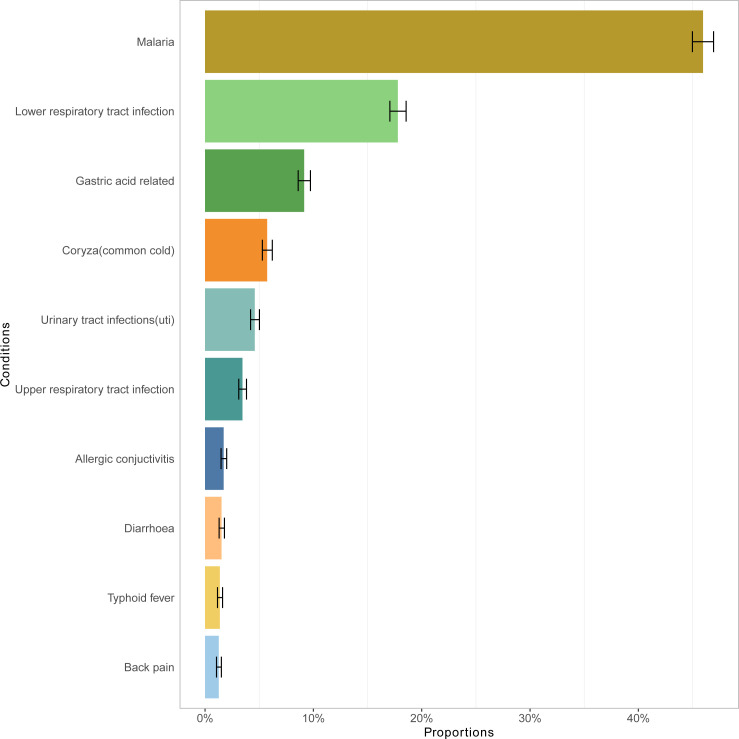
The top 10 diagnosed diseases in the Iganga-Mayuge HDSS population for the time period 2018–2023. HDSS, Health and Demographic Surveillance Site.

In the entire study population, malaria, lower respiratory tract infection, coryza (common cold), gastric acid-related and urinary tract infection (UTI) ranked in the first 5 places and accounted for over 83% of all the diagnosed diseases, with percentages of 45.97% (95% CI 44.99%, 46.94%), 17.79% (95% CI 17.06%, 18.56%), 9.15% (95% CI 8.60%, 9.73%), 5.73% (95% CI 5.29%, 6.21%) and 4.59% (95% CI 4.20%, 5.02%), respectively (figure 1).

**Figure 2:**
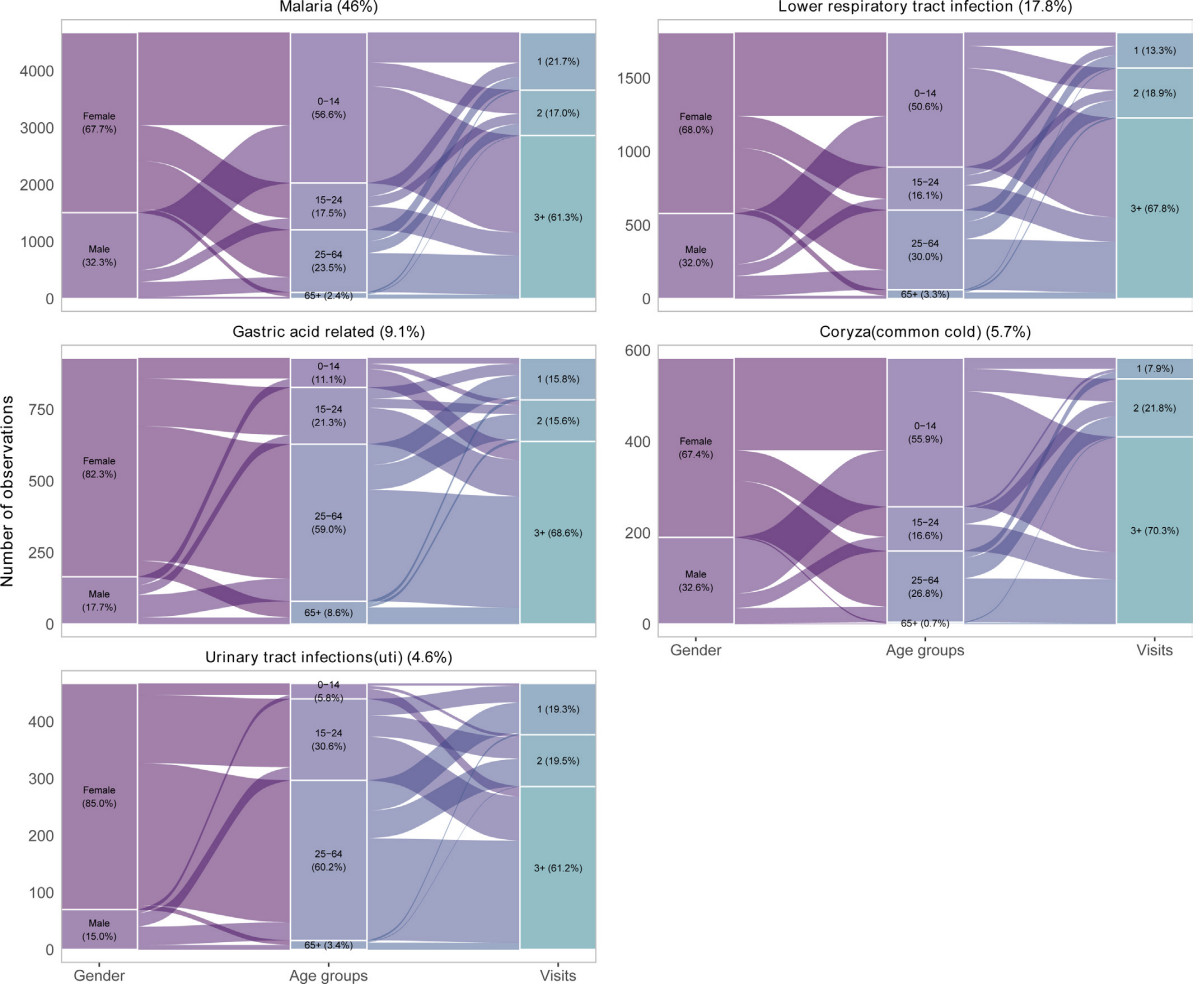
Top five leading diagnosed diseases by various demographic factors.

As shown in figure 2, in all the top five diagnosed diseases, more females were diagnosed with the diseases compared with their male counterparts, accounting for over 60% in each of the diagnosis. While malaria (56.6%), lower respiratory tract infections (50.6%) and coryza (55.9%) were mostly common in children aged 0–14 years, gastric acid-related (59.0%) diseases and UTIs (60.2%) were common in adults aged 25–64 years. Among all the diagnosed malaria cases, over 60% had visited the health facility at least three times, the majority of whom were female. Similar patterns were observed in other diagnosed diseases.

**Figure 3:**
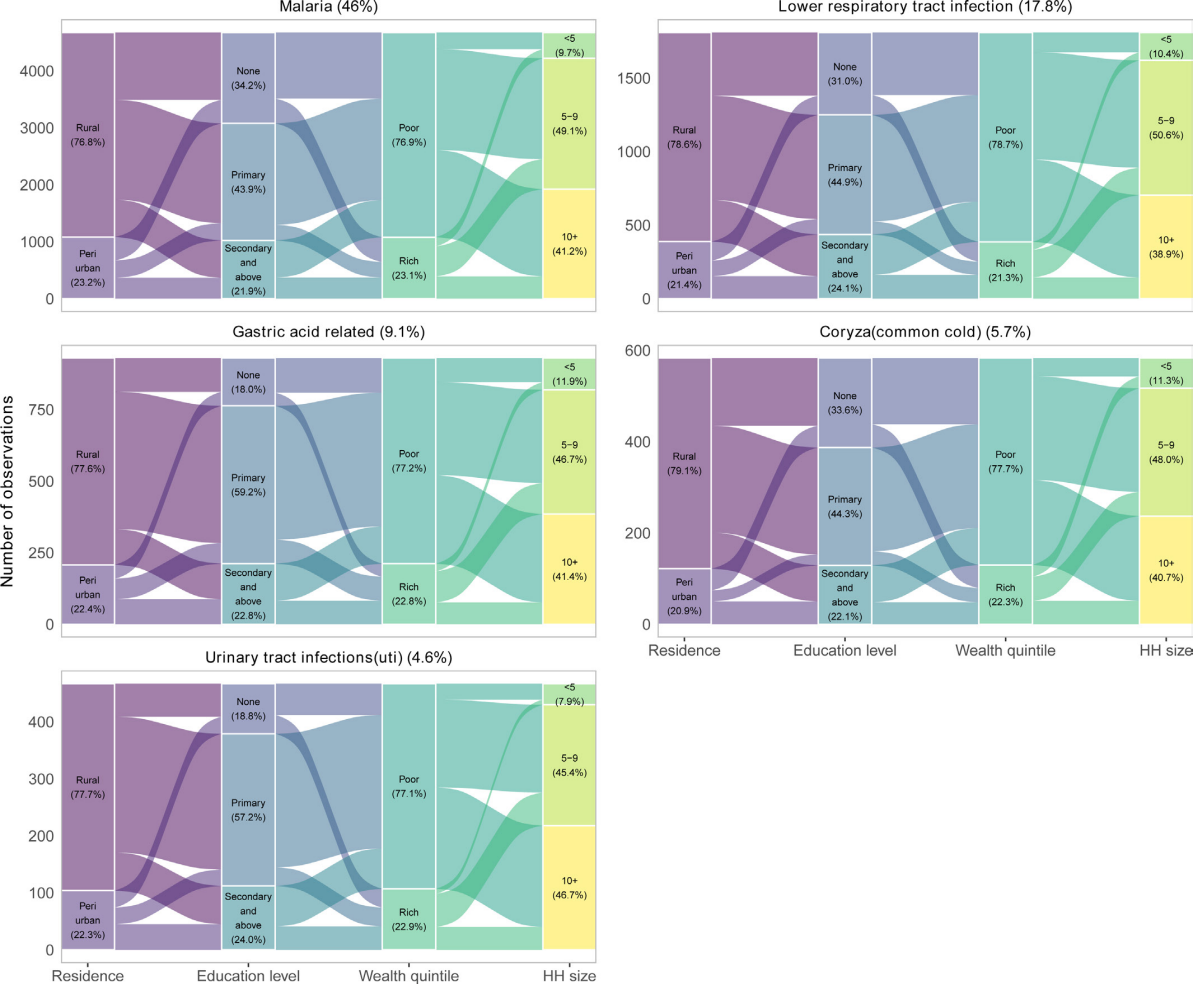
Top five leading diagnosed diseases by various demographic and socioeconomic factors for patients who had socioeconomic information collected within the HDSS. HDSS, Health and Demographic Surveillance Site.

Figure 3 compares the top five diagnosed diseases across various socioeconomic factors. In all the top five diagnosed conditions, most patients were from rural areas, malaria (76.8%), lower respiratory tract infection (78.6%), gastric acid-related (77.6%), coryza (79.1%) and UTIs (77.7%), and the majority either had no formal education or had only achieved primary education. In terms of wealth status, poor patients were primarily from rural in comparison to rich patients, who were mainly from periurban areas. Large households were mostly from rural areas in comparison to those from small households.

### Demographic and socioeconomic determinants of morbidity

Table 2 presents the regression coefficients for the mixed-effects multivariate multinomial logistic model fitted to the data for diagnosed conditions, including malaria, gastric acid-related disorders, lower respiratory tract infections and UTIs, with coryza used as the reference category. Generally, most predictors did not show clear differences in the risk of being diagnosed with a particular disease. However, for gender, male patients had a significantly lower risk of being diagnosed with gastric acid-related diseases (relative odds ratio (ROR) 0.59, 95% CI 0.45 to 0.78) and UTIs (ROR 0.48, 95% CI 0.34 to 0.68) compared with female patients. For age groups, compared with children aged 0–14 years, patients aged 15–24 years had a significantly higher risk of being diagnosed with gastric acid-related disorders (ROR 6.02, 95% CI 4.23 to 8.62) and UTIs (ROR 16.60, 95% CI 10.00 to 27.93). Patients aged 25–64 years also showed significantly higher risk for gastric acid-related diseases (ROR 10.46, 95% CI 7.65 to 14.54) and UTIs (ROR 21.68, 95% CI 13.56 to 34.70). Elderly patients (65+ years) had a significantly higher risk for all conditions, with particularly elevated risk for gastric acid-related diseases (ROR 64.51, 95% CI 24.97 to 220.07) and UTIs (ROR 52.39, 95% CI 16.91 to 218.74). For the number of visits, patients with two visits had a significantly lower risk of being diagnosed with all conditions compared with those with only one visit, with relative risk of 0.29 (95% CI 0.20 to 0.40) for malaria, 0.35 (95% CI 0.23 to 0.54) for gastric acid-related diseases, 0.51 (95% CI 0.35 to 0.73) for lower respiratory tract infections and 0.34 (95% CI 0.22 to 0.53) for UTIs. Similar trends were observed for patients with three or more visits. For year of diagnosis, compared with the year 2018, the risk of being diagnosed with all conditions significantly decreased over the years, particularly for lower respiratory tract infections, with the relative risk dropping to 0.54 (95% CI 0.33 to 0.85) in 2021 and further in subsequent years. Significant reductions were also noted for other conditions in the later years, especially in 2022 and 2023. The random effects component indicates a variance ($\sigma^{2}$) of 0.03 for the patient-specific correlation and an intraclass correlation of 0.03, reflecting the proportion of total variance explained by patient-specific differences.

**Table 2:** Estimates from the mixed-effects multivariate multinomial logistic model fitted to the data for diagnosed conditions—malaria, gastric acid-related, lower respiratory tract infection, urinary tract infection and coryza (reference category)

Predictors	Malaria	Gastric acid related	Lower respiratory tract infection	Urinary tract infection
	ROR* (95% CI†)	ROR (95% CI)	ROR (95% CI)	ROR (95% CI)
Intercept	25.97 (15.27 to 46.44)	0.80 (0.40 to 1.63)	6.68 (3.80 to 12.24)	0.16 (0.06 to 0.41)
Gender				
Female‡	1.00	1.00	1.00	1.00
Male	0.92 (0.76 to 1.12)	**0.59†† (0.45 to 0.78)**	0.98 (0.80 to 1.22)	**0.48 (0.34 to 0.68)**
Age groups (years)				
0–14‡	1.00	1.00	1.00	1.00
15–24	0.97 (0.76 to 1.24)	**6.02 (4.23 to 8.62)**	1.10 (0.83 to 1.45)	**16.60 (10.00 to 27.93)**
25–64	0.86 (0.69 to 1.07)	**10.46 (7.65 to 14.54)**	1.25 (0.98 to 1.58)	**21.68 (13.56 to 34.70)**
65+	**3.44 (1.45 to 12.68)**	**64.51 (24.97 to 240.07)**	**5.20 (2.12 to 19.08)**	**52.39 (16.91 to 218.74)**
Education level				
None‡	1.00	1.00	1.00	1.00
Primary	0.98 (0.80 to 1.21)	1.30 (0.97 to 1.75)	1.01 (0.80 to 1.26)	1.14 (0.80 to 1.63)
Secondary and above	0.94 (0.74 to 1.22)	1.06 (0.75 to 1.50)	1.09 (0.83 to 1.44)	0.99 (0.65 to 1.49)
Residence				
Rural‡	1.00	1.00	1.00	1.00
Periurban	1.16 (0.88 to 1.52)	1.17 (0.81 to 1.67)	1.24 (0.93 to 1.66)	0.97 (0.63 to 1.47)
Wealth status				
Poor‡	1.00	1.00	1.00	1.00
Rich	0.95 (0.74 to 1.24)	1.15 (0.82 to 1.62)	0.89 (0.68 to 1.19)	1.07 (0.70 to 1.63)
Household size				
*<*5‡	1.00	1.00	1.00	1.00
5–9	1.23 (0.91 to 1.64)	1.13 (0.77 to 1.66)	1.21 (0.87 to 1.68)	1.61 (0.98 to 2.64)
10+	1.26 (0.94 to 1.70)	1.26 (0.84 to 1.86)	1.11 (0.80 to 1.55)	**1.86 (1.14 to 3.13)**
Number of visits				
1‡	1.00	1.00	1.00	1.00
2	**0.29 (0.20 to 0.40)**	**0.35 (0.23 to 0.54)**	**0.51 (0.35 to 0.73)**	**0.34 (0.22 to 0.53)**
3+	**0.30 (0.22 to 0.41)**	**0.46 (0.31 to 0.65)**	**0.54 (0.37 to 0.74)**	**0.33 (0.22 to 0.49)**
Year of diagnosis				
2018‡	1.00	1.00	1.00	1.00
2019	0.90 (0.56 to 1.36)	0.81 (0.47 to 1.34)	0.94 (0.57 to 1.47)	1.05 (0.55 to 1.92)
2020	0.93 (0.60 to 1.38)	0.72 (0.43 to 1.17)	0.76 (0.48 to 1.18)	0.75 (0.42 to 1.35)
2021	0.80 (0.51 to 1.21)	0.61 (0.35 to 1.02)	**0.54 (0.33 to 0.85)**	0.59 (0.32 to 1.09)
2022	**0.54 (0.35 to 0.80)**	**0.36 (0.21 to 0.60)**	**0.40 (0.25 to 0.62)**	0.59 (0.32 to 1.06)
2023	0.64 (0.38 to 1.05)	**0.35 (0.18 to 0.67)**	**0.36 (0.20 to 0.60)**	0.52 (0.25 to 1.11)
Random effects				
*σ*2	0.03			
*τ* 00	0.96			
ICC*§*	0.03			
Unique observations¶	3489			
Observations**	8462			

* Relative Odds Ratioodds ratio.

† Confidence IntervalConfidence interval.

‡ Reference category.

§ Intra-class Ccorrelation.

¶ Number patients who had top 5five conditions.

** Total number of visits made by patients who reported top 5five conditions.

†† Bolded numbers indicate demographic and socioeconomic factors that significantly influenced a particular morbidity

We also include the expected probability of being diagnosed with a particular disease conditioned on a particular level of a predictor of interest—the conditional effect plots in online supplemental material 2, figure 1.

## Discussion

The central aim of this study was to gain an in-depth understanding of the implications of demographic and socioeconomic differences on disease burden in Uganda. We analysed unique and first-of-its-kind data that links community-level and household-level data with health facility records within a selected study community in Eastern Uganda—the Iganga-Mayuge HDSS. Our results provide evidence of demographic and socioeconomic differences in the burden of disease among the studied population between 2018 and 2023. We applied a mixed-effects multinomial logistic model to analyse different combinations of morbidity of malaria, lower respiratory tract infection, gastric and related diseases, UTI and coryza (common cold). The multinomial logistic regression model accommodates the complexity of morbidity patterns and provides a framework for understanding the simultaneous presence or absence of multiple diseases in individuals, reflecting the real-world epidemiological overlap.^31^

The most prevalent diagnosed conditions were malaria, lower respiratory tract infections, coryza (common cold), gastric acid-related issues and UTIs. Collectively, these top five health conditions represented over 83% of all diagnosed diseases, with malaria leading at ~46% and across all demographic and socioeconomic factors. The disease prevalence pattern found in this study is comparable to the national patterns where data are available, with malaria accounting for 48.3%. The patterns of commonly diagnosed conditions found in this study are also similar to those observed in most SSA countries outside of Uganda.^32^

The observed higher proportions of women visiting health clinics compared with men across all diagnosed conditions reflect significant disparities in care-seeking behaviours between genders, as observed in.^33 34^ This discrepancy is attributed to cultural stigmas that discourage men from seeking healthcare,^35^,^37^ particularly for infectious diseases.^38^,^40^ Notably, men often defer seeking care until faced with life-threatening situations,^41^,^43^ contributing to elevated morbidity rates and diminished life expectancy.^44^ Addressing these cultural and gender-specific barriers is pivotal to promoting equitable access to healthcare, mitigating morbidity and improving overall health outcomes within the population. Efforts to destigmatise healthcare-seeking behaviours among men may yield substantial benefits in reducing the burden of preventable illnesses and enhancing the well-being of the community.

More than 50% of the sample comprised children and adolescents, a proportion consistent with the expected high number based on the region’s demographics.^20^ This indicates a notable tendency among parents to prioritise their children’s hospital visits, potentially at the expense of their own health.^45^ The majority of the patients had no formal education, with those possessing postprimary education making up less than 25%. Although this percentage is low, it aligns with expectations. Uganda implemented universal free primary education in 1997, but a significant number of children still do not attend school. Considering the portion of the sample aged 5 and under who are not yet expected to be in formal education, the 31% without education appears reasonable.^46^

The observed variations in disease patterns highlight the disparities prevalent in this region. On the whole, there were no notable differences in disease burden based on the area of residence. While it is widely recognised globally and in SSA that individuals in rural areas face higher morbidity and mortality rates,^47^,^50^ this comparison typically involves urban residents rather than those in periurban settings, as seen in the sample. In rural Uganda, there is a heavier reliance on private practitioners, as government health clinics are predominantly concentrated in urban communities.^51^ Given that individuals in periurban areas must also travel to urban centres for enhanced healthcare access, it can be inferred that increased investments in government-funded health clinics in non-urban areas would contribute to improved population health.^51^

Attaining postsecondary education also proved to be a non-significant protective factor against malaria and UTIs. While higher education is generally linked to improvements in overall morbidity and mortality rates,^25 26^ this was not clearly reflected in our specific findings. This scenario presents an opportunity for further growth and subsequent advancements in addressing health disparities.^52^ Initiatives to enhance access to higher education should begin with overall improvements in education throughout Uganda.^53^ Despite persistent cost barriers for primary education and ongoing challenges in inclusive education access for girls, rural students and those with disabilities,^54^ the potential health benefits of increased education make it crucial to address these barriers. Although external scholarship programmes exist and contribute to improved access and outcomes for participants, their limited and unequal access does not fully address nationwide disparities.^55^

Surprisingly, wealth status demonstrated minimal influence on disease burden. Individuals in the highest income bracket exhibited a slightly lower risk for infectious diseases in the sample and a higher risk of gastric diseases, although the risk displayed wide variation and lacked clear distinctions. The lack of clarity of these findings might be attributed to marginal differences in income tertiles within this sample. Additionally, income status may not significantly enhance healthcare accessibility, given the specific location of health centres within the Iganga-Mayuge HDSS. Education, recognised as a significant determinant of health, could exert a more pronounced effect due to heightened health education and improved access to urban centres, offering expanded options for both education and healthcare.

Gastric acid-related problems tend to increase with age.^56^ Conditions such as gastro-oesophageal reflux disease (GERD)^57^,^59^ and peptic ulcers are more commonly observed in individuals over the age of 65. While occasional acid reflux is common in children,^60^,^62^ serious diagnoses of gastrointestinal (GI) issues are rare in this age group.^63^ GI diseases, especially GERD, exhibit a higher prevalence among females.^64 65^ Female sex hormones reduce oesophageal pressure, contributing to increased GI symptoms.^66^ The significant impact of age and gender on the treatment of gastric acid-related illnesses in this sample indicates that interventions and policies aimed at improving GI health should focus on older adult women.

The analysis revealed significant disparities in UTI diagnoses based on gender and age, as observed in the literature.^67^,^69^ Possible hypothesis for gender differences is that females are at a higher risk due to anatomical differences, such as a shorter urethra and proximity to the anus, as well as hormonal changes that occur during pregnancy and menopause.^67^ These physiological factors necessitate gender-specific public health interventions aimed at improving hygiene practices and promoting timely healthcare access for women, especially in resource-limited settings such as Iganga-Mayuge HDSS. On the other hand, the risk of UTI increases markedly with age, particularly among older adults.^68 69^ This could be driven by factors such as a weakening immune system, hormonal shifts in postmenopausal women and the presence of chronic conditions.^68^ The increasing incidence of UTIs with age highlights the need for enhanced infection control measures, particularly in geriatric care, including regular screening and prompt treatment.

Age did not demonstrate a significant impact on malaria in this sample, which was unexpected given that malaria typically affects children more than adults.^70^ However, due to the substantial proportion of the sample being under the age of 20, there might not have been sufficient statistical power to establish significance. This is supported by online supplemental material 2, figure 1, illustrating a decline in the probability of malaria as age increases. It is crucial to highlight that individuals aged 14 years and below constituted a majority of the malaria visits. Hence, it is plausible that a monotonic relationship exists and, as such, cannot be adequately represented by the assumed linear effect of age.

A higher number of visits showed a significant protective effect on the probability of being diagnosed with any of the conditions. This is attributed to increased utilisation of healthcare services, including preventative measures. Most of these conditions are easily treatable with minimal visits^71^ and involve fewer diagnostic and treatment complexities compared with other illnesses in this sample.^9 23^ It is also plausible that individuals with a greater frequency of visits to the health clinic have been long-term residents of the Iganga-Mayuge HDSS, making them more proficient in preventing and self-treating some of the endemics.

There are several strengths to this study. It included a large sample size with a demographic breakdown that matches the region. Our findings are, therefore, generalisable to non-urban regions in Uganda, and understandings from this health facility can be applied to other Uganda health facilities. The use of an objective measure of disease burden such as healthcare utilisation with an official diagnosis reduces the bias and provides a comparable metric of morbidity. Finally, the use of longitudinal data makes the analysis more robust and provides insight into trends in disease burden over the 6-year period.

### Limitations

This study has several limitations. The analysis was restricted to the top five disease diagnoses, resulting in an incomplete representation of the full burden of less common illnesses. Additionally, there was no inclusion of a control group without any diagnosed condition for comparison. Lastly, the endemic prevalence of malaria in Iganga, contributing to its heightened burden of disease, might limit the generalisability of the findings to regions with a low prevalence of malaria.

## Conclusion

Our study highlights the potential to use existing data sources at both community and health facility levels to assess disease burden in a rural African setting. By using health and demographic surveillance sites to emphasise health utilisation and its variations based on demographic and socioeconomic variables, we provide a foundation for policymakers to formulate strategies aimed at improving population health. The persistence of endemic diseases, such as malaria, despite receiving substantial funding over the years, highlights the existing gap between the community population and policy-makers. To address this, policy-makers should formulate targeted policies that guide health prevention and promotion efforts, particularly for common and recurrent diseases such as malaria. Continuous assessment of the epidemiological landscape and the effectiveness of existing interventions should inform the shift towards more targeted and innovative strategies. Additionally, community engagement and strengthening health systems play a pivotal role in ensuring sustained impact. By leveraging the lessons learnt from past initiatives and staying responsive to evolving challenges, endemic countries such as Uganda can foster resilience in their malaria control efforts and contribute to the global goal of reducing the burden of this deadly disease.

## supplementary material

10.1136/bmjph-2024-000898 online supplemental file 1

## Data Availability

Data are available on reasonable request.
