# Supplementary material for: Understanding the demographic and socioeconomic determinants of morbidity in Eastern Uganda: a retrospective analysis of the Iganga-Mayuge health and demographic surveillance data
Source: BMJ Public Health. 2024 Nov 28;2(2):e000898. doi: 10.1136/bmjph-2024-000898 (PMC11816860; doi:10.1136/bmjph-2024-000898)
Supplement: online supplemental file 1 [file bmjph-2-2-s001.pdf]

Supplemental Material 1: Statistical model

To understand the population and social determinants of morbidity, we fitted a mixed effects multivariate multinomial logistic model. This model allows us to investigate the relationship between multiple diagnosed conditions (malaria, gastric acid-related conditions, lower respiratory tract infections, urinary tract infections, coryza (common cold)) and various demographic and socioeconomic factors such as age groups, education level, wealth status, household size, number of visits to the facility and year of diagnosis, with coryza (common cold) as the reference category. This statistical approach allows for the simultaneous comparison of the odds of different diagnosed conditions relative to the reference category while accounting for multiple demographic and socioeconomic factors (predictor) [1]. The patient-specific, relating to a particular visit to the health facility, was included to account for the correlation due to multiple visits, possible multiple diagnosis.

Let $\mathrm{Y_{ijk}}$ and $\mathrm{\pi_{ijk}}$ be the diagnosed disease status and probability of being diagnosed with a particular disease, respectively, i.e., malaria ($\mathrm{k} = 1$), lower respiratory tract infection ($\mathrm{k} = 2$), gastric acid related ($\mathrm{k} = 3$), urinary tract infection ($k = 4$), and coryza (common cold) ($\mathrm{k} = 5$) of patient $\mathrm{i} = 1, \cdots,$ in visit $\mathrm{j} = 1, \cdots,$. We assume that $\mathrm{Y_{ijk}}$ follows a multinomial distribution, i.e., $\mathrm{Y_{ijk}} \sim \mathrm{MN}(1, \pi_{\mathrm{ijk}})$, where $\pi_{\mathrm{ijk}} = (\pi_{\mathrm{ij1}}, \cdots, \pi_{\mathrm{ij5}})^{\mathrm{T}}$ is the probability of patient i being diagnosed with condition $\mathrm{k} \in 1, \cdots, 5$ in visit j. The probability of being diagnosed with a particular disease, $\mathrm{k} \in 1, \cdots, 4$, relative to coryza (common cold) ($k = 5$), in visit j is defined as

$$\pi_{ijk} = \frac{\eta_{ijk}}{1 + \sum_{l=1}^{k} \exp(\eta_{ijl})}, \qquad k = 1,2,3,4$$

where η_{ijk} is the linear predictor given as

$$\eta_{ijk} = \beta_{0k} + \beta_{1k} \cdot \text{age group}_{ij} + \beta_{2k} \cdot \text{education level}_{ij} + \beta_{3k} \cdot \text{wealth status}_{ij} + \beta_{4k} \cdot \text{household size}_{ij} + \beta_{5k} \cdot \text{number of visits}_{ij} + \beta_{6k} \cdot \text{year of diagnosis}_{ij} + \tau_{ik}$$

and τ_{ik} is patient-specific random effects accounting for the correlation due to repeated measurements. For the reference category, $\mathrm{k} = 5$, $\pi_{\mathrm{ij5}} = 1 - \sum_{\mathrm{k}=1}^{4} \pi_{\mathrm{ijk}}$.

More specifically, the linear predictors for each of the diagnosis category, other than Coryza, are specified as follows:

Malaria:

$$\eta_{ij,\mathrm{Malaria}} = \beta_{0,\mathrm{Malaria}} + \beta_{1,\mathrm{Malaria}} \cdot \text{age group}_{ij} + \beta_{2,\mathrm{Malaria}} \cdot \text{education level}_{ij} + \beta_{3,\mathrm{Malaria}} \cdot \text{wealth status}_{ij} + \beta_{4,\mathrm{Malaria}} \cdot \text{household size}_{ij} + \beta_{5,\mathrm{Malaria}} \cdot \text{number of visits}_{ij} + \beta_{6,\mathrm{Malaria}} \cdot \text{year of diagnosis}_{ij} + \tau_{i,\mathrm{Malaria}}$$

Lower Respiratory Tract Infection (LRTI):

$$\eta_{ij,\mathrm{LRTI}} = \beta_{0,\mathrm{LRTI}} + \beta_{1,\mathrm{LRTI}} \cdot \text{age group}_{ij} + \beta_{2,\mathrm{LRTI}} \cdot \text{education level}_{ij} + \beta_{3,\mathrm{LRTI}} \cdot \text{wealth status}_{ij} + \beta_{4,\mathrm{LRTI}} \cdot \text{household size}_{ij} + \beta_{5,\mathrm{LRTI}} \cdot \text{number of visits}_{ij} + \beta_{6,\mathrm{LRTI}} \cdot \text{year of diagnosis}_{ij} + \tau_{i,\mathrm{LRTI}}$$

Gastric Acid Related (GAR):

$$\eta_{ij,\mathrm{GAR}} = \beta_{0,\mathrm{GAR}} + \beta_{1,\mathrm{GAR}} \cdot \text{age group}_{ij} + \beta_{2,\mathrm{GAR}} \cdot \text{education level}_{ij} + \beta_{3,\mathrm{GAR}} \cdot \text{wealth status}_{ij} + \beta_{4,\mathrm{GAR}} \cdot \text{household size}_{ij} + \beta_{5,\mathrm{GAR}} \cdot \text{number of visits}_{ij} + \beta_{6,\mathrm{GAR}} \cdot \text{year of diagnosis}_{ij} + \tau_{i,\mathrm{GAR}}$$

Urinary Track Infection (UTI):

$$\eta_{ij,\mathrm{UTI}} = \beta_{0,\mathrm{UTI}} + \beta_{1,\mathrm{UTI}} \cdot \text{age group}_{ij} + \beta_{2,\mathrm{UTI}} \cdot \text{education level}_{ij} + \beta_{3,\mathrm{UTI}} \cdot \text{wealth status}_{ij} + \beta_{4,\mathrm{UTI}} \cdot \text{household size}_{ij} + \beta_{5,\mathrm{UTI}} \cdot \text{number of visits}_{ij} + \beta_{6,\mathrm{UTI}} \cdot \text{year of diagnosis}_{ij} + \tau_{i,\mathrm{UTI}}$$

Each coefficient $(\beta_{\mathrm{m,k}})$ (for $(\mathrm{m} = 0,1, \ldots, 6)$) represents the effect of the corresponding predictor variable on the log-odds of diagnosis k relative to Coryza. The conditional probability of each diagnosis category (other than Coryza) is given by:

Malaria:

$$P\left(Y_{ij} = \text{Malaria}\right) = \frac{\exp\left(\eta_{ij,\text{Malaria}}\right)}{1 + \exp\left(\eta_{ij,\text{Malaria}}\right) + \exp\left(\eta_{ij,\text{LRTI}}\right) + \exp\left(\eta_{ij,\text{GAR}}\right) + \exp\left(\eta_{ij,\text{UTI}}\right)}$$

Lower Respiratory Tract Infection (LRTI):

$$P\left(Y_{ij} = \text{LRTI}\right) = \frac{\exp\left(\eta_{ij,\text{LRTI}}\right)}{1 + \exp\left(\eta_{ij,\text{Malaria}}\right) + \exp\left(\eta_{ij,\text{LRTI}}\right) + \exp\left(\eta_{ij,\text{GAR}}\right) + \exp\left(\eta_{ij,\text{UTI}}\right)}$$

Gastric Acid Related (GAR):

$$P\left(Y_{ij} = \text{GAR}\right) = \frac{\exp\left(\eta_{ij,\text{GAR}}\right)}{1 + \exp\left(\eta_{ij,\text{Malaria}}\right) + \exp\left(\eta_{ij,\text{LRTI}}\right) + \exp\left(\eta_{ij,\text{GAR}}\right) + \exp\left(\eta_{ij,\text{UTI}}\right)}$$

Urinary Track Infection (UTI):

$$\mathrm{P}\left(\mathrm{Y}_{\mathrm{ij}} = \mathrm{UTI}\right) = \frac{\exp\left(\eta_{\mathrm{ij,UTI}}\right)}{1 + \exp\left(\eta_{\mathrm{ij,Malaria}}\right) + \exp\left(\eta_{\mathrm{ij,LRTI}}\right) + \exp\left(\eta_{\mathrm{ij,GAR}}\right) + \exp\left(\eta_{\mathrm{ij,UTI}}\right)}$$

For the reference category (**Coryza**), the probability is:

$$P\left(Y_{ij} = \text{Coryza}\right) = \frac{1}{1 + \exp\left(\eta_{ij,\text{Malaria}}\right) + \exp\left(\eta_{ij,\text{LRTI}}\right) + \exp\left(\eta_{ij,\text{GAR}}\right) + \exp\left(\eta_{ij,\text{UTI}}\right)}.$$

All analyses were performed using R software. The R package brms [2] was used to fit the model.

Supplemental Material 2: Conditional effect plot

Fig. 1 shows the expected probability of being diagnosed with a particular disease conditioned on a particular level of a predictor of interest - the conditional effect plots. Furthermore, it shows how the predicted probabilities differ across various levels of the predictor. Other than age groups and number of visits, in which the probability of being diagnosed with Malaria is higher in children aged 0-14 years and those patients who had one visit, respectively, the expected probability of being diagnosed with a particular condition remained relatively the same across different levels of the predictor of interest. Furthermore, the probability of being diagnosed with coryza is higher in older patients. On the other hand, the probability of being diagnosed with lower respiratory tract infection has decreased since 2018. This is not the case with Malaria.

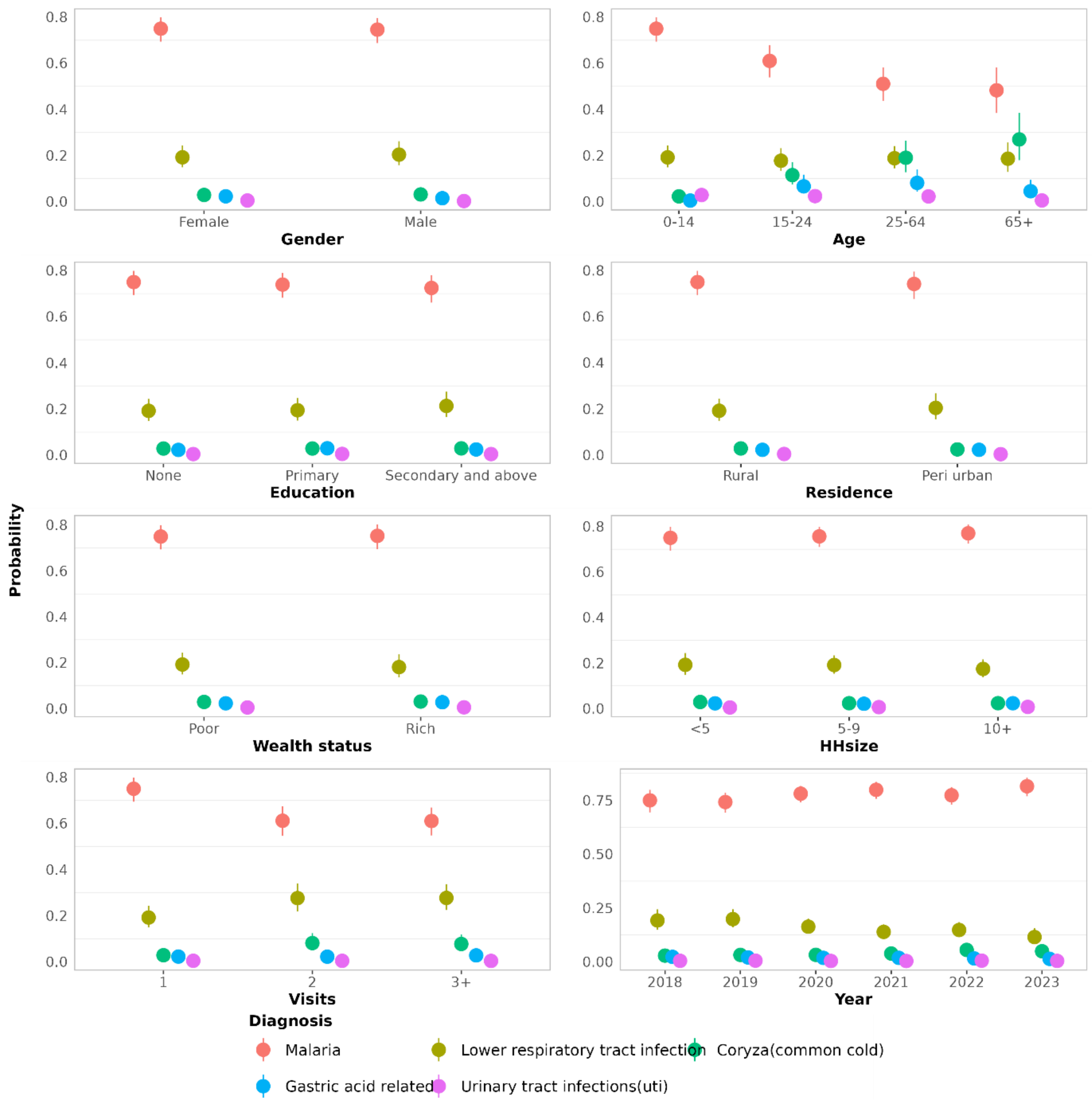

Fig. 1: A comparison of the conditional effect plots for all predictors.

References

1. Kazembe LN, Namangale JJ. A Bayesian multinomial model to analyse spatial patterns of childhood co-morbidity in Malawi. Eur J Epidemiol 2007;22(8):545–56.

2. Bürkner P-C. **brms** : An *R* Package for Bayesian Multilevel Models Using *Stan*. J Stat Softw [Internet] 2017 [cited 2023 Nov 23];80(1). Available from: http://www.jstatsoft.org/v80/i01/
